# Investigating associations between physical activity, stress experience, and affective wellbeing during an examination period using experience sampling and accelerometry

**DOI:** 10.1038/s41598-023-35987-8

**Published:** 2023-05-31

**Authors:** Justin Hachenberger, Ziwen Teuber, Yu-Mei Li, Laura Abkai, Elke Wild, Sakari Lemola

**Affiliations:** 1 Department of Psychology, Bielefeld University, Universitätsstraße 25, 33615 Bielefeld, Germany; 2 Department of Behavioural and Cognitive Sciences, University of Luxembourg, Esch-sur-Alzette, Luxembourg; 3 Department of Psychology, University of Warwick, Coventry, United Kingdom

**Keywords:** Psychology, Risk factors

## Abstract

Previous studies reported that physical activity could buffer the negative association of psychological stress with affective wellbeing. However, the studies that examined this relation in everyday life have assessed physical activity only by self-report but not with objective measures such as accelerometry. We therefore investigated the associations of both subjectively and objectively measured physical activity with stress experiences and affective wellbeing. A total of 90 university students participated in a 10-day experience sampling and diary study during their examination period and reported about stress experiences, physical activity, and affective states. Physical activity was additionally assessed using accelerometry in 50 of the participants. Subjectively assessed physical activity and objectively assessed light physical activity were associated with feeling less stressed in the evening. Also, light physical activity during the day was associated with a smaller increase/higher decrease in feeling stressed from morning to evening. The association of stress experience with negative affect was moderated by objective light physical activity. No interactive effects of stress intensity and physical activity on affective wellbeing were found. On stressful days, physical activity may buffer the negative association between stress and affective wellbeing. Particularly light physical activity as assessed with accelerometry seems to play an important role. It may be beneficial for students' affective wellbeing to increase or at least maintain physical activity during examination periods.

## Introduction

Psychosocial stress has been linked to various health-related problems including coronary heart disease, systemic inflammation, obesity, depression, and anxiety^1–3^. On an acute level, stress can have a negative impact on general wellbeing and affective states^4–8^. The reactivity to stressors seems to be of particular importance as heightened negative affective reactivity to stressors has been linked to the increased probability of the onset of affective disorders and chronic physical health conditions^9,10^. In addition to the possible reduction of exposure to stressors, it would also be beneficial to identify behavioural factors that mitigate stress reactivity. Physical activity has been discussed to be one of these behaviours. Regular physical activity is associated with better health^11,12^ and acute physical activity is associated with subsequent elevated mood^13–19^. Moreover, individuals who engaged in physical activity during the day felt less stressed in the evening^20^.

Following this notion, physical activity seemed to buffer the negative impacts of stress. The cross-stressor-adaptation hypothesis^21,22^ posits that physical activity is a stressor itself and that regular engagement in physical activity builds up adaptive resources that also reduce the reactivity to stressors in other domains (e.g., work or study related stress). This is supported by numerous experimental/laboratory studies^23–25^ comparing the stress reactivity in individuals with low vs. high habitual physical activity. For example, Rimmele et al.^24^ induced a stressor in a laboratory setting to male athletes and to untrained adult males. While individuals of both groups showed an increase in anxiety and a decrease in calmness, the effects were less pronounced in the athletes. Furthermore, von Haaren et al.^26,27^ applied an aerobic exercise intervention on sedentary students and found that individuals in the intervention group showed less stress reactivity of the autonomous nervous system and less emotional reactivity to a stressor than individuals in the control group. The effects described in the cross-stressor-adaptation hypothesis rather refer to the effects of habitual physical activity (trait-level) that bolster against stress in a generalized manner and not to acute effects (state-level) of engaging in physical activity on the risk of experiencing stress afterwards.

On an acute level, laboratory studies by Bernstein and McNally^28,29^ found that individuals who performed a 30-min session of aerobic exercise were impacted less and recovered faster emotionally from subsequent stressors than those who rested for 30 min. This is in line with the stress-buffering hypothesis^30^, which posits that physical activity attenuates the adverse effects of stress (i.e., on daily affect), and which is applicable to both regular and acute physical activity^31,32^. However, most studies testing the stress-buffering effects of physical activity on affect relied on artificially (laboratory) induced stressors or focused on analysing between-individual variation which, contrary to the long-held assumption, do not necessarily reflect within-individual processes^33,34^. Within-individual processes and daily dynamics can be investigated with intensive longitudinal data by employing an experience sampling methodology (ESM) to capture participants’ experiences in natural settings with great ecological validity^35^.

To the best of our knowledge, only two studies have investigated day-to-day variations of physical activity, experienced stress, and affective wellbeing^36,37^. Puterman et al.^36^ assessed daily engagement in physical activity, exposure to stressful events, and negative affect for eight consecutive days and found that negative affect was elevated on days with stressful events, but that this association was attenuated when participants were active on the corresponding day. Flueckiger et al.^37^ investigated students across an entire academic year and found that the more physically active an individual was on a given day, the weaker the association between stress and affect. However, these interaction effects were relatively small. One limitation of these two studies is that physical activity was assessed solely by self-reports.

The present study aims to investigate the associations of physical activity, stress, and affective wellbeing in daily life by also considering objectively assessed physical activity. We therefore applied an ESM design with additional diary methods and an ambulatory assessment of physical activity with accelerometers. In particular, we focused on university students during a high-stakes examination period. During examination periods, students experience a high level of psychosocial stress particularly if the examination determines their future academic or career trajectories^38^. Previous studies on real life stress have examined such examination periods because exams are considered to be naturally occurring stressors^18,26,27,39^. Based on the rationale described above, we tested the following hypotheses:

- (H1a) Higher subjective/objective physical activity throughout the day is associated with lower stress in the evening.
- (H1b) Higher subjective/objective physical activity throughout the day is associated with a smaller increase or larger decrease in feeling stressed across the day (stress measured in the evenings minus stress measured in the mornings).
- (H2a) Days with stressful events are associated with lower levels of positive affect/higher levels of negative affect, whereas this association is moderated by subjective/objective physical activity on the same day.
- (H2b) On days with stressful events, higher stress intensity is followed by lower positive affect/higher negative affect, whereas this association is moderated by subjective/objective physical activity on the same day.

All hypotheses were investigated separately under consideration of different types/intensities of physical activity (light physical activity, LPA; moderate physical activity, MPA; vigorous physical activity, VPA; moderate-to-vigorous physical activity MVPA) as previous studies have mainly focused on MVPA alone^36,37^. Furthermore, we explored whether the association described in hypotheses (1a/b) were moderated by the number of days remaining before the exam (i.e., testing whether the stress increased as the exam approached).

## Methods

### Procedure

This study was conducted in accordance with the Declaration of Helsinki and with approval of the Ethics Committee of Bielefeld University (reference number 2021-282-Am). The participants were recruited at Bielefeld University via the study participation management system of the Department of Psychology, announcements during lectures of psychology students, poster advertisements at Bielefeld University, and word-of-mouth advertisements among the students. To sign up for the study, the participants filled out an online questionnaire in which they were first informed about the procedures and conditions of the study, data handling (including their legal rights regarding data protection and withdrawal from the study), and the conditions for receiving compensation. All participants provided informed consent. Furthermore, the participant provided information about the date of their first exam in the upcoming examination period. In addition to taking an exam, having a smartphone with an Android operating system was also required. Participants also had the option of borrowing a lab phone for the duration of the study participation. Furthermore, participants could choose whether they want to wear an accelerometer for the duration of the data collection.

The data collection took place during the examination periods of the winter term 2021/22 (February/March 2022) and the summer term 2022 (July/August 2022). However, the exact period of data collection was tailored specifically for each participant depending on their first exam date. Eleven days before their first exam, the participants completed a baseline questionnaire (approx. 15 min) in which information about demographics and the upcoming exam, sleep, and physical activity habits, and somatic, anxiety and depressive symptoms were collected. On the next day, experience sampling questionnaires (including some diary questions) presented on movisensXS (version 1.5.23; library version 7365; movisens GmbH, Karlsruhe, Germany) started. Participants received two short questionnaires (approx. 2 min)—one in the mornings and one in the evenings. The prompt for the morning questionnaire was sent out at 9:00 a.m. on weekdays and at 10:00 a.m. on weekends. The morning questionnaire assessed participants’ affective states, information about their sleep in the previous night, and their current achievement goals concerning their studies. The prompt for the evening questionnaire was sent out at 7:00 p.m. on all days. The evening questionnaire assessed participants’ affective states, stressful experiences and physical activity during the day, and their achievement emotions concerning their studies. Participants could respond to the questionnaire within 60 min after receiving each prompt. If participants did not respond within 60 min, this questionnaire was marked as missing. Participants were instructed to ignore prompts in situations that could cause danger to themselves or other (e.g., while driving).

Participants who agreed on additionally wearing an accelerometer (GENEActiv, Activinsights Ltd., Kimbolton, UK) for the duration of the study were instructed to wear the accelerometer on their non-dominant wrist starting at 6:00 p.m. on the day they completed the baseline questionnaire (11 days before their exam) until waking up on the day of their exam. Participants were informed that they could take off the accelerometer if a situation required it (e.g., when showering or doing certain sports). However, participants were encouraged to wear it as much as possible.

Participants who completed at least 80% of all questionnaires received either a 15€ voucher or 3 research participation credits. Two 50€ vouchers were raffled among all students qualifying for compensation. Participants who agreed on additionally wearing an accelerometer received an additional 15€ voucher if they wore the accelerometer for at least 80% of the required time.

### Participants

In total, 101 participants signed up for the study. However, 9 participants did not start the study (e.g., because they at some point decided not to take the exam anymore or due to technical incompatibilities) and 2 participants provided fewer than two measurements (see Statistical analysis). Thus, the final sample consisted of 90 participants (71 females, 17 males, and 2 participants who preferred not to report their gender) with a mean age of 24.0 (*SD* 4.4). Among them, 50 participants agreed on additionally wearing an accelerometer. There were no significant differences between the participants who agreed on additionally wearing an accelerometer and participants, who did not, regarding any study variable (see Table 1).

**Table 1:** Sample demographics and descriptive statistics.

	Full sample (*N* = 90)		Accelerometry		
			No (*n* = 40)	Yes (*n* = 50)	*t* (*df*)
	*M* (*SD*)/*n* (%)	Min–Max	*M* (*SD*)/*n* (%)	*M* (*SD*)/*n* (%)	
**Demographics**					
Age	24.0 (4.4)	18.0–40.0	24.4 (5.1)	23.6 (3.8)	0.74 (70.1)^a^
Gender					
Prefer not to answer	2.0 (2.2)		1.0 (2.5)	1.0 (2.0)	
Female	71.0 (78.9)		32.0 (80.0)	39.0 (78.0)	
Male	17.0 (18.9)		7.0 (17.5)	10.0 (20.0)	
**Diary/ESM measures**					
Available evening measurements	8.3 (2.0)	1.0–10.0	8.2 (2.2)	8.4 (1.9)	− 0.35 (78.0)^a^
*Affective wellbeing*					
Feeling stressed (morning)	48.2 (21.3)	0.3–100.0	50.5 (24.5)	46.3 (18.4)	0.89 (70.6)^a^
Feeling stressed (evening)	48.4 (21.3)	3.9–99.9	50.6 (24.2)	46.6 (18.7)	0.86 (72.0)^a^
Difference in feeling stressed	− 0.2 (10.6)	− 30.9–24.3	− 0.7 (8.8)	0.2 (12.0)	− 0.41 (85.5)^a^
Days with a stressful experience	3.9 (2.6)	0.0–10.0	4.1 (2.6)	3.8 (2.6)	0.50 (82.5)^a^
Stress intensity	60.6 (15.6)	23.0–100.0	63.0 (17.6)	58.8 (13.7)	1.18 (65.0)^a^
Positive affect (morning)	143.2 (43.9)	1.8–234.0	135.0 (44.0)	149.8 (43.1)	− 1.61 (83.0)^a^
Positive affect (evening)	145.0 (45.5)	5.0–223.2	138.6 (48.6)	150.0 (42.7)	− 1.16 (78.3)^a^
Negative affect (morning)	108.9 (62.5)	1.2–292.8	123.7 (72.4)	97.0 (51.1)	1.97 (67.8)^a^
Negative affect (evening)	110.7 (61.8)	6.3–286.7	119.2 (70.7)	103.9 (53.5)	1.13 (71.0)^a^
*Physical activity*					
MVPA (h)	1.1 (1.5)	0.0–11.1	1.3 (1.9)	0.9 (1.0)	0.93 (55.3)^a^
VPA (h)	0.4 (0.7)	0.0–3.7	0.4 (0.7)	0.4 (0.7)	1.40 (48.8)^a^
MPA (h)	0.7 (0.9)	0.0–7.5	0.8 (1.3)	0.5 (0.5)	0.06 (81.8)^a^
**Accelerometry**					
ACC-MVPA (h)	1.1 (0.5)	0.0–3.7	–	1.1 (0.5)	–
ACC-VPA (h)	0.0 (0.1)	0.0–0.7	–	0.0 (0.1)	–
ACC-MPA (h)	1.0 (0.5)	0.0–3.7	–	1.0 (0.5)	–
ACC-LPA (h)	1.5 (0.5)	0.0–2.7	–	1.5 (0.5)	–
Sedentary behaviour (h)	8.2 (1.1)	4.8–10.6	–	8.2 (1.1)	–
ACC-ENMO (m*g*)	36.6 (12.5)	3.4–91.8	–	36.6 (12.5)	–

*MVPA* moderate-vigorous physical activity (sum of VPA and MPA), *VPA* vigorous physical activity, *MPA* moderate physical activity, *LPA* light physical activity, *ENMO* Euclidean norm minus one. Positive affect sum score is computed from the items content, happy, and enthusiastic. Negative affect sum score is computed from the items afraid, sad, and worried. The descriptive statistics about the duration of physical activity are daily averages.

^a^No significant difference between participants without vs. with accelerometry was found.

### Measures and instruments

#### Physical activity

##### Questionnaire-assessed physical activity

The subjective physical activity measures were assessed in the evening questionnaires. Participants were asked to indicate how long (in hours and minutes) they had engaged in MPA (“How long did you engage in moderate physical activity today [e.g., carrying light things, riding a bicycle at a normal speed, or playing light sports]?”) and VPA (“How long did you engage in vigorous physical activity [e.g., strenuous physical work or strenuous sports such as riding a bike fast, playing soccer, or jogging] today?”) that day. These items were adopted from the International Physical Activity Questionnaire (see http://ipaq.ki.se)^40^, but modified for daily assessment. The durations of MPA and VPA were summed to calculate the total duration of MVPA of each day until participants started answering the questionnaire.

#### Accelerometer-assessed physical activity

The objective physical activity measures were derived from the raw acceleration data measured by triaxial accelerometers (GENEActiv, Activinsights Ltd., Kimbolton, UK). A sampling rate of 60 Hz was used. The raw data was processed with the R-package *GGIR* (version 2.8-0)^41^ applying auto-calibration^42^. A configuration file containing the applied settings of the GGIR function can be found under (osf.io/ybd9w). The raw triaxial acceleration signals were transformed to the Euclidean Norm Minus One (ENMO; expressed in milli gravitational units m*g*) which is an omnidirectional metric used to quantify acceleration of movement^43,44^. Here, the gravitational acceleration (1 g = 9.81 m/s^2^) is subtracted from the vector magnitude of the three axes to correct for gravity. Negative values are rounded up to 0. The ENMO metric was further aggregated into 1-min epochs. Each 1-min epoch was then categorised into the following physical activity intensities based on the ENMO cut-off values^45^ proposed by Hildebrandt et al.^46,47^: sedentary (< 45.8 mg); LPA (45.8–93.2 mg), MPA (93.2–418.3 mg), and VPA (≥ 418.3 mg). Based on this categorisation, the duration (in hours) of sedentary behaviour, LPA, MPA, VPA, MVPA (sum of MPA and VPA), and the mean ENMO from waking up to completing the evening questionnaire was calculated for each participant for the days when they completed the evening questionnaires. The time of waking up was derived by using the sleep detection implemented in GGIR^48,49^. The prefix “ACC” is used to indicate the accelerometer-assessed physical activity variables in the rest of the article.

#### Stress experience

In the morning and evening questionnaires, participants were asked to indicate how stressed they currently feel (“How stressed do you feel at the moment?”; 0 = *not at all*, 100 = *very much*). The difference in feeling stressed between the morning and evening measurements was calculated by subtracting the value of the morning measurement from the evening measurement.

In the evening questionnaire, participants were also asked whether they had experienced a stressful event or if they felt stressed during the day (“Were you stressed or exposed to a stressful event during the day today?”; 0 = *no*, 1 = *yes*). If the participants responded “yes”, they were further asked how intense the stress was in this context (“How intense was the stress?”; 0 = *not at all*, 100 = *very much*).

#### Affective states

Similar to Das-Friebel et al.^50^, positive affect was measured with three positive items including “content”, “enthusiastic”, and “happy”, whereas negative affect was measured with three negative items including “afraid”, “sad”, and “worried”. Participants were asked to indicate on a visual analogue scale to which extent they felt each affective state (“How … do you feel at the moment?”; 0 = *not at all*, 100 = *very much*). The items were presented in a randomised order. Internal consistency was found to be good for both, positive (α = 0.85) and negative affect (α = 0.85). Sum scores for positive and negative affect were computed. Higher sum scores indicate higher levels of positive and negative affect.

### Statistical analysis

Data pre-processing and all statistical analyses were performed with R (version 4.2.1)^51^. The analysis plan was preregistered on Open Science Framework (osf.io/rgdve). The changes of the analysis plan are described in Supplement 1. Multilevel models computed with the R-package *lme4* (version 1.1-30)^52^ were used to test the hypotheses. For the analyses of the main hypotheses, all physical activity variables (*subjective*: MVPA, VPA, MPA; *objective*: ACC-MVPA, ACC-VPA, ACC-MPA, ACC-LPA, ACC-ENMO) were used as predictors in separate models.

To test hypotheses 1a and b, each physical activity variable was included as a predictor while feeling stressed in the evening and the difference in feeling stressed (evening measurement minus morning measurement) were included as the outcome variable, respectively. In additional exploratory models, the variable indicating the number of days before the exam (1 = 10 days before the exam, 10 = 1 day before the exam) with an interaction term with physical activity was added. We further examined whether and to which degree ACC-LPA and ACC-MVPA differed in how they were associated with stress experience in hypotheses H1a/b. For this purpose, we employed isotemporal substitution models^53^ using the duration (in hours) of ACC-LPA, ACC-MVPA, and total duration of all activities (total duration of all activities is defined as the sum of ACC-sedentary behaviour, ACC-LPA, and ACC-MVPA) per day as predictors of the respective outcomes of hypotheses 1a/b. By excluding the duration of ACC-sedentary behaviour as a predictor but considering it for the computation of total duration of activities, the remaining coefficients of ACC-LPA and ACC-MVPA represent the consequence of substituting one hour of sedentary behaviour with 1 h of the respective intensity of physical activity.

To test hypothesis 2a, a dichotomous variable of whether there was a stressful experience on the respective day (*no*/*yes*; reference = *no*), each physical activity variable (in separate models), and an interaction term were included as predictors. Additionally, we calculated corresponding simple slopes with the R-package *interactions* (version 1.1.0)^54^ to examine the association of physical activity and affective wellbeing on days with and without stressful experiences. To test hypothesis 2b, the stress intensity of a stressful experience, each physical activity variable (in separate models), and an interaction term were included as predictors. Positive and negative affect sum scores were used as outcome variables separately in models testing hypotheses 2a/b.

All numerical variables were within-individual standardised (using the individual mean and standard deviation of each participant) before the analysis to facilitate interpretation and the comparison of effect sizes as well as to account for within-individual effects. An exception to that are the predictors used in the isotemporal substitution models, for which the original unit of measurement (hours) was retained. Maximum likelihood estimation was used to obtain parameters of the multilevel models. A significance threshold of α = 0.05 was used. To address the problem of alpha error accumulation in multiple comparisons, all *p*-values were adjusted using false discovery rate (FDR)^55^.

## Results

### Descriptive statistics

The demographic characteristics and descriptive statistics are shown in Table 1. Overall, participants provided 1508 of 1800 possible responses (on average 16.8, *SD* 3.7, per participant) resulting in an overall completion rate of 83.8%, suggesting good overall compliance. For the evening questionnaire alone, 749 responses were provided (on average 8.3, *SD* 2.0, per participant).

### Within-individual associations of physical activity and feeling stressed

Multilevel models testing hypothesis 1a revealed that more subjective MVPA (β = − 0.16, *p* < 0.001), VPA (β = − 0.15, *p* < 0.01), and MPA (β = − 0.13, *p* < 0.01) during the day were followed by feeling less stressed in the evening. Concerning objective physical activity measures, only more ACC-LPA (β = − 0.14, *p* < 0.05) was followed by feeling less stressed in the evening (Supplementary Table 1). No interactions between subjective/objective physical activity measures and days before the upcoming exam were found. The results of the isotemporal substitution model suggest that it is more beneficial to replace one hour of sedentary time during the day with ACC-LPA (β = − 0.22, *p* < 0.05) than with ACC-MVPA (β = 0.02, *p* = 0.890).

Multilevel models testing hypothesis 1b showed that more ACC-LPA was associated with a smaller increase (or larger decrease, respectively) in feeling stressed from the mornings to the evenings (β = − 0.15, *p* < 0.05). No other subjective/objective physical activity measures were associated with the difference in feeling stressed from the mornings to the evenings (Supplementary Table 2). Also, no interactions between subjective/objective physical activity measures and the variable representing the number of days remaining before the exam were found. The results of the isotemporal substitution model suggest that it is more beneficial to replace one hour of sedentary time during the day with ACC-LPA (β = − 0.20, *p.unadj* < 0.05, *p.adj* = 0.059) rather than with ACC-MVPA (β = 0.05, *p* = 0.689) although this association was not statistically significant after accounting for multiple testing.

### Differences in the association of physical activity with affective states on days with and without a stressful experience

#### Positive affect

**Figure 1:**
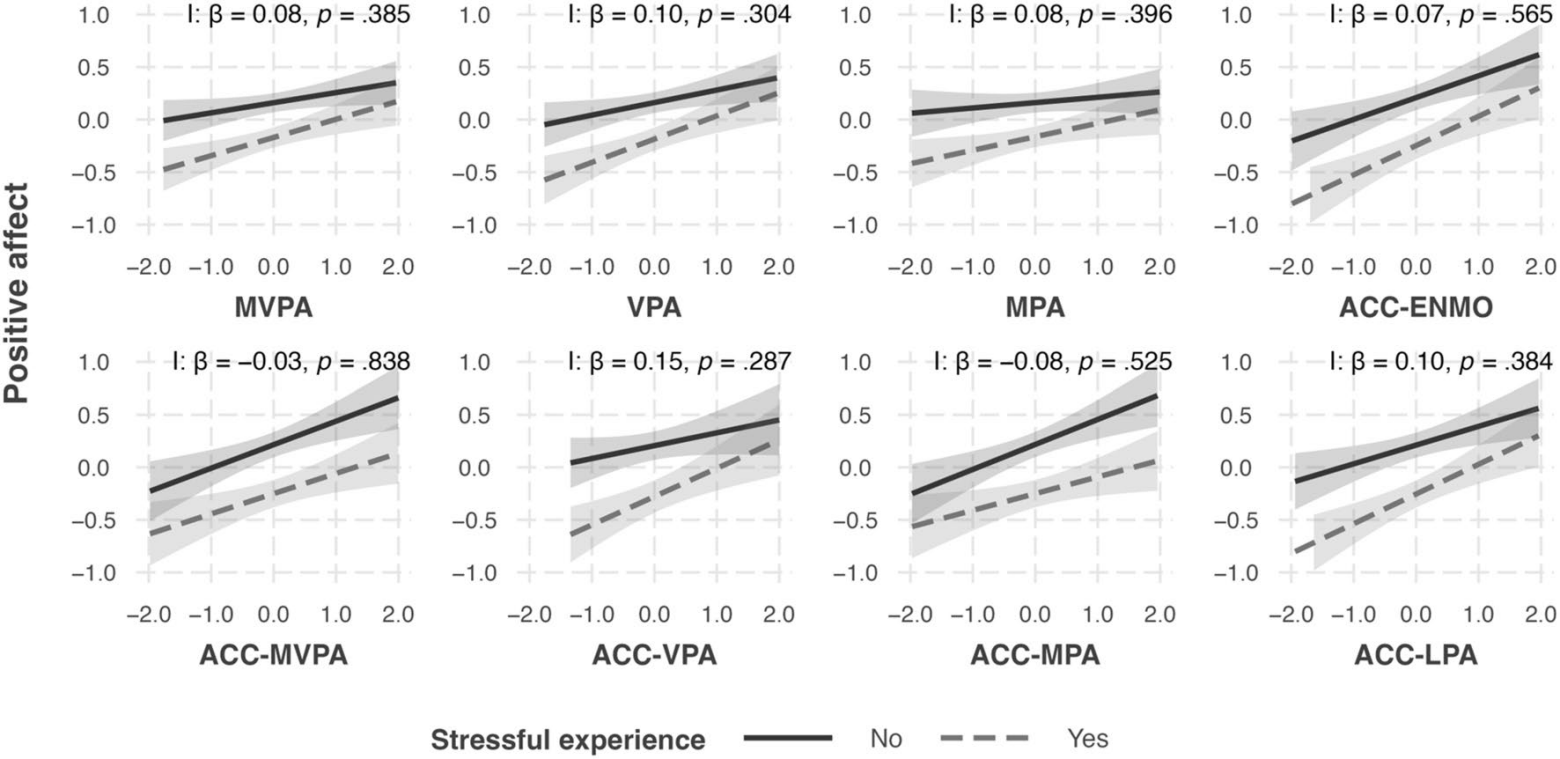
Simple slopes of the associations of physical activity measures with positive affect on days with vs. without a stressful experience. *I* interaction, *MVPA* moderate-vigorous physical activity, *VPA* vigorous physical activity, *MPA* moderate physical activity, *LPA* light physical activity, *ENMO* Euclidean norm minus one, *ACC* accelerometry. *p < 0.05 before FDR-adjustment.

Multilevel models testing hypothesis 2a revealed that days with a stressful experience were consistently associated with lower positive affect in the evening compared to days without a stressful experience (Supplementary Table 3). The associations of subjective/objective physical activity measures with positive affect were not moderated by the occurrence of a stressful experience during the day. An investigation of the corresponding simple slopes (see Fig. 1 and Supplementary Table 5) showed that the objective physical activity measures were consistently associated with higher positive affect (except for ACC-VPA on days without a stressful experience), while subjective physical activity measures were only associated with higher positive affect on days with stressful experiences.

#### Negative affect

**Figure 2:**
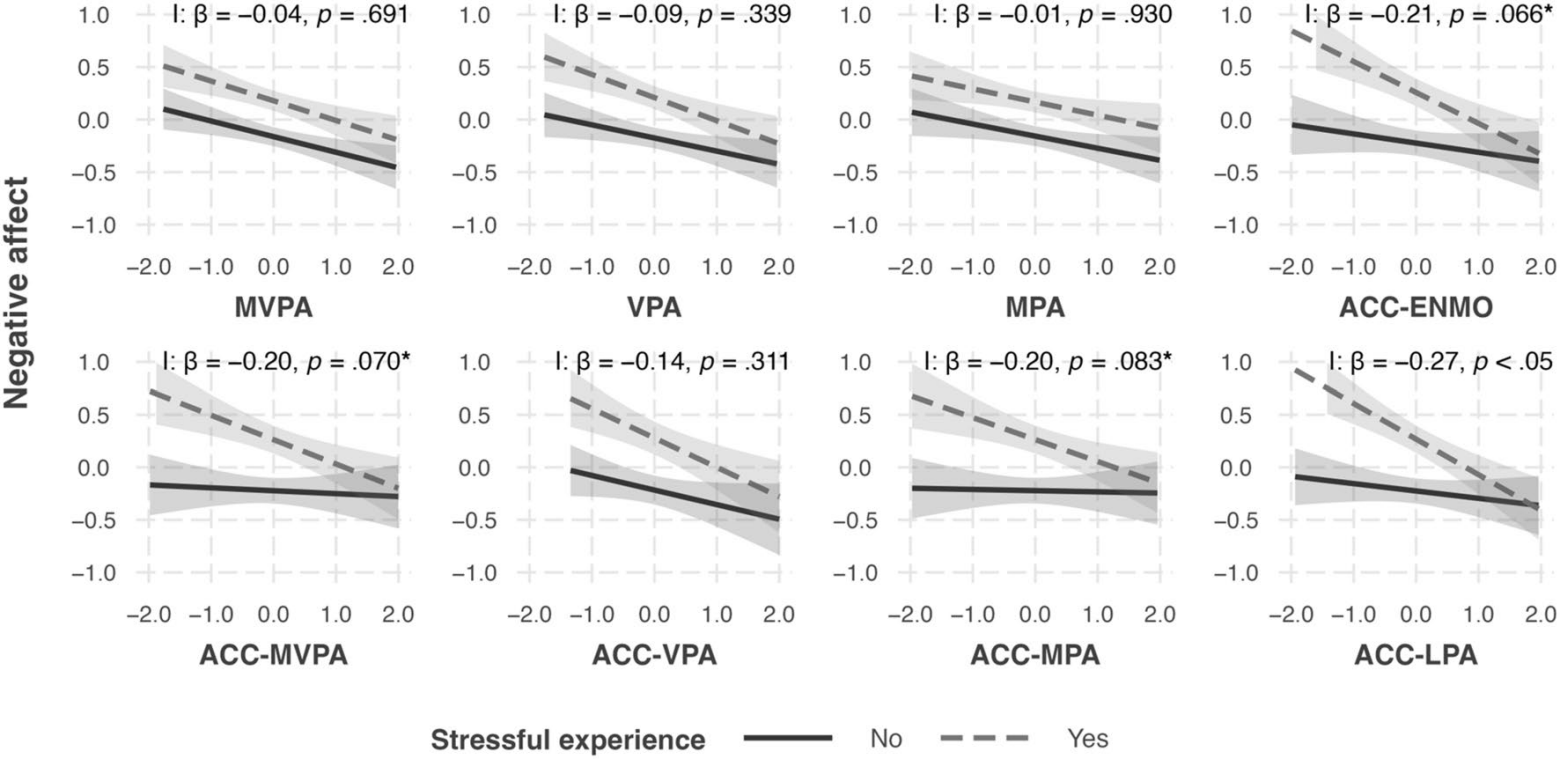
Simple slopes of the associations of physical activity measures with negative affect on days with vs. without a stressful experience. *I* interaction, *MVPA* moderate-vigorous physical activity, *VPA* vigorous physical activity, *MPA* moderate physical activity, *LPA* light physical activity, *ENMO* Euclidean norm minus one, *ACC* accelerometry. *p < 0.05 before FDR-adjustment.

Multilevel models testing hypothesis 2a revealed that days with a stressful experience were consistently associated with higher negative affect in the evening compared to days without a stressful experience (Supplementary Table 4). The association of ACC-LPA with negative affect was moderated by the occurrence of a stressful experience during the day (β = − 0.27, *p* < 0.05). Before FDR-adjustment, the associations of ACC-MVPA (β = − 0.20, *p.unadj* < 0.05, *p.adj* = 0.070), ACC-MPA (β = − 0.20, *p.unadj* < 0.05, *p.adj* = 0.083), and ACC-ENMO (β = − 0.21, *p.unadj* < 0.05, *p.adj* = 0.066) with negative affect were also moderated by the occurrence of a stressful experience during the day. An investigation of the corresponding simple slopes (see Fig. 2 and Supplementary Table 5) showed that the subjective physical activity measures were consistently associated with lower negative affect, except for MPA on days without a stressful experience. By contrast, objective physical activity measures were consistently associated with lower negative affect only on days with a stressful experience.

### Interactive effects of physical activity and stress intensity on days with a stressful experience

#### Positive affect

Multilevel models testing hypothesis 2b revealed that on days with a stressful experience, higher stress intensity was consistently associated with lower positive affect in the evening. No interaction between stress intensity and subjective/objective physical activity measures was found. However, we found significant main effects of physical activity measures with positive affect, indicating that more subjective/objective physical activity throughout the day was associated with higher positive affect in the evening (except for MPA and ACC-MPA). All results are displayed in Supplementary Table 6.

#### Negative affect

Multilevel models testing hypothesis 2b revealed that on days with a stressful experience, higher stress intensity was consistently associated with higher negative affect in the evening. No interaction between stress intensity and subjective/objective physical activity measures was found. However, we found significant main effects of physical activity measures with negative affect, indicating that more physical activity throughout the day was associated with lower negative affect in the evening (except for MPA). All results are displayed in Supplementary Table 7.

## Discussion

There are four key findings of the present research. First, we found that all subjectively assessed physical activity variables were associated with feeling less stressed in the evening, while concerning the objectively assessed physical activity variables, an association was only found for LPA (Hypothesis 1a). In this regard, we also showed that substituting one hour of ACC-sedentary time with ACC-LPA appeared to be more beneficial in terms of feeling less stressed in the evening than substituting it with ACC-MVPA. Second, we found that only ACC-LPA was associated with a smaller increase (or larger decrease, respectively) in feeling stressed from mornings to evenings (Hypothesis 1b). Third, we found that the experience of stress was associated with lower positive affect and higher negative affect and that more objective physical activity during the day was generally associated with higher positive and lower negative affect in the evening. However, only the association of stress experience with negative affect was moderated by ACC-LPA (Hypothesis 2a). Fourth, we did not find interactive effects of physical activity and stress intensity on affective states (Hypothesis 2b).

Our finding that subjective physical activity was associated with feeling less stressed is generally consistent with results from previous studies^18,20^. However, these studies have neither differentiated between physical activity intensities nor investigated objective physical activity measures. Concerning subjectively assessed physical activity, MVPA was associated with feeling less stressed which is comparable to the results by Schultchen et al.^18^. However, concerning objectively assessed physical activity we only found that LPA was associated with feeling less stressed, but not MVPA or MPA and VPA individually. This discrepancy between subjectively and objectively assessed physical activity might be explained by the notion that participants either overestimated the intensity of their physical activity in the self-reports or that the accelerometry did not assess or classify the intensity of physical activity adequately. Some studies have shown that people tend to overestimate the duration or intensity of physical activity in self-reports compared to accelerometry^56,57^.

Whereas past studies have found interactive effects of physical activity and stress on affective wellbeing which support the idea that physical activity could buffer negative impacts of stress^36,37^, we found inconclusive support for this claim in the present study. The two studies by Flueckiger et al.^37^ and Puterman et al.^36^ differed in the way stress and physical activity were assessed. While Flueckiger et al.^37^ analysed stress intensity and physical activity of a day as continuous variables, Puterman et al.^36^ focused on the occurrence of a stressful event and the performance of physical activity as dichotomous variables (no vs. yes). In our analyses, treating the occurrence of a stressful experience as a dichotomous variable as done by Puterman et al.^36^, we showed that the association of stress experience with negative affect was moderated by ACC-LPA in such a way that more ACC-LPA mitigated the negative association of stress experience and negative affect. A similar pattern was also observed for objectively measured MPA, MVPA and ENMO, which, however, was attenuated after FDR-adjustment. These results are consistent with predictions of the stress-buffering hypothesis^30–32^. In contrast, when considering stress intensity as a continuum as has been done by Flueckiger et al.^37^, we did not find interactive effects of stress intensity and physical activity on affective wellbeing. Flueckiger et al.^37^ reported that the interactive effects were rather small in their study as it is common when day-to-day fluctuations are investigated. In the present study, we may have had too little power to detect these small effects due to a limited number of participants and measurements.

Despite not being the main focus of the present study, two further results merit commenting. First, we consistently found physical activity to be associated with lower negative affect on days with stressful events, but not on days without stressful events. Previous studies found inconsistent results concerning the association of physical activity with lower negative affect, while the evidence for the association of physical activity with higher positive affect are rather consistent^15–19^. Our results suggest that these inconsistent findings by other studies may be explained by the circumstance that—in the sense of the stress-buffering hypothesis^30^—physical activity may only mitigate negative affect if a specific stressor occurred that elicits negative affect. This is further supported by one of the few studies that was also conducted during an examination period which found evidence that more physical activity is associated with lower negative affect^18^ implying an elevated stress experience for university students^38^. Second, there is limited evidence on how long the supposed beneficial effects of physical activity on affective wellbeing last. Some studies that investigated this aspect found evidence that acute effects of physical activity on affective states last up to approximately 3 h^16,19^, while another study found that the mean physical activity of a day is not associated with next day’s affect anymore^58^. While in the present study we did not examine the role of the time between the performance of physical activity and the measurement of affect, it suggests that the whole day’s average of physical activity was associated with affective states in the evening.

Overall, our results highlight the beneficial associations of physical activity with affective states and stress experience. LPA in particular seems to be of relevance. If replicated with experimental designs, these findings yield important implications including that it is recommendable for students to stay physically active during examination periods. Hereby, our findings are in line with the notion that it is particularly beneficial, to maintain or incorporate LPA (e.g., going for a walk) into everyday life to offset the negative impacts of this stressful time and to improve affective wellbeing.

Although the present results generally support the notion of stress-buffering effects of physical activity, it is necessary to be aware of several limitations. First, we adopted an observational design which does not allow any causal conclusions. It is therefore possible that days with increase of LPA were different from other days in that students were generally less focused on preparing the exams but spending more leisure time which involved LPA. In order to pin down the causal relationship between physical activity and later affect, an experimental design has to be employed. Second, the generalisability of the results is limited as we focused on university students and the sample consisted mainly of young adult female participants. Third, we did not consider the exact timing of physical activity or the occurrence of a stressful event. The sequence of occurrence of the stressful event and the performance of the physical activity as well as the time interval between them could play a role^59^. Fourth, the outcome variables affect and stress were assessed based on momentary states, which could, however, be influenced by contextual factors (e.g., current company, environment, or activity) of the respective situation that we did not collect or control for. Finally, we did not consider the domain in which physical activity was performed (i.e., leisure-time or work-related physical activity), which appears to be an important factor^15^.

In terms of future research, apart from addressing the limitations mentioned above (i.e., using experimental/intervention designs and studying samples drawn from other age, gender, and social background-related groups), it would be important to extend the current findings by examining the role of LPA in more detail as previous research has mostly focused on MVPA. Furthermore, it would be important to focus on physiological aspects of the interrelation of physical activity and stress experience. For example, heart rate variability as a measure of vagal cardiac control, which is interrelated with physical activity, affective wellbeing, and stress, could be focused^26,60^.

## Conclusion

We found that subjective physical activity and objective LPA were associated with feeling less stressed. Moreover, more objective LPA moderated the association of stress experience with negative affect which is in line with predictions of the stress-buffering hypothesis. Overall, our results suggest that LPA in particular might be of paramount relevance.

## Supplementary Information

Supplementary Information.

## Data Availability

The datasets used during the current study are available from the corresponding author on reasonable request.
