# Supplementary material for: Investigating associations between physical activity, stress experience, and affective wellbeing during an examination period using experience sampling and accelerometry
Source: Sci Rep. 2023 May 31;13:8808. doi: 10.1038/s41598-023-35987-8 (PMC10232510; doi:10.1038/s41598-023-35987-8)
Supplement: Supplementary file 1 — Supplementary Information. [file 41598_2023_35987_MOESM1_ESM.pdf]

Supplementary information

Investigating associations between physical activity, stress experience, and affective wellbeing during an examination period using experience sampling and accelerometry

Justin Hachenberger[*1], Ziwen Teuber[2], Yu-Mei Li[1], Laura Abkai[1], Elke Wild[1], Sakari Lemola[1,3]

[1]Department of Psychology, Bielefeld University, Bielefeld, Germany

[2]Department of Behavioural and Cognitive Sciences, University of Luxembourg

[3]Department of Psychology, University of Warwick, Coventry, United Kingdom

Supplement 1

We made four changes to the analysis plan that is preregistered on the Open Science Framework (https://osf.io/qpmje):

1. Originally, we planned to analyse the metabolic equivalent of task of physical activity intensities. However, we decided to merely focus on the durations.
2. Originally, we planned to only analyse moderate and vigorous physical activity assessed with accelerometry. However, we decided to also include light physical activity to complement the spectrum of physical activity intensities. Furthermore, we included the ENMO (Euclidean norm minus one) metric as a continuous, non-categorised variable indicating overall movement/physical activity.
3. For hypotheses 1a/b, we originally planned to only analyse each intensity of physical activity in separate multilevel models. However, we also computed isotemporal substitution models in which light physical activity, moderate-to-vigorous physical activity, and total activity time were included as predictors so that we can eventually compare the effects of each intensity in contrast to sedentary behaviour (see explanation in the main text).
4. We originally planned to only investigate the interactive effects of intensity of stressful events with physical activity on affective states (originally hypothesis 2, now hypothesis 2b; similar to Flueckiger et al., 2016). However, we decided to also contrast days with vs. without stressful events (hypothesis 2a; similar to Puterman et al., 2017).

Supplementary Table 1

Results of multilevel models testing the associations of physical activity variables with feeling stressed in the evening (H1a)

Predictor	Beta	*SE*	*df*	*t*	*p.unadj*	*p*
MVPA	-0.16	0.04	736	-4.37	< .001	< .001
VPA	-0.15	0.04	590	-3.61	< .001	< .01
MPA	-0.13	0.04	693	-3.49	< .001	< .01
ACC-MVPA	-0.10	0.05	396	-1.97	< .05	.092
ACC-VPA	-0.07	0.06	297	-1.24	.217	.320
ACC-MPA	-0.08	0.05	396	-1.64	.102	.173
ACC-LPA	-0.14	0.05	396	-2.72	< .01	< .05
ACC-ENMO	-0.11	0.05	396	-2.25	< .05	.056

Note. MVPA moderate-vigorous physical activity; VPA vigorous physical activity; MPA moderate physical activity; LPA light physical activity; ENMO Euclidean norm minus one; ACC accelerometery; *p.unadj* unadjusted *p*-value.

Supplementary Table 2

Results of multilevel models testing the associations of physical activity variables with the difference in feeling stressed from morning to evening (H1b)

Predictor	Beta	*SE*	*df*	*t*	*p.unadj*	*p*
MVPA	-0.07	0.04	637	-1.79	.073	.132
VPA	-0.09	0.04	526	-2.11	< .05	.068
MPA	-0.06	0.04	606	-1.57	.116	.191
ACC-MVPA	-0.11	0.05	342	-2.13	< .05	.066
ACC-VPA	-0.01	0.06	259	-0.21	.831	.900
ACC-MPA	-0.11	0.05	342	-2.14	< .05	.066
ACC-LPA	-0.15	0.05	342	-2.89	< .01	< .05
ACC-ENMO	-0.09	0.05	342	-1.77	.078	.136

Note. MVPA moderate-vigorous physical activity; VPA vigorous physical activity; MPA moderate physical activity; LPA light physical activity; ENMO Euclidean norm minus one; ACC accelerometery; *p.unadj* unadjusted *p*-value.

Supplementary Table 3

Results of multilevel models testing the associations of the occurrence of a stressful experience during the day, various physical activity variables, and an interaction of both with positive affect in the evening

	Predictor	Beta	*SE*	*df*	*t*	*p.unadj*	*p*
MVPA	SE	-0.33	0.07	736	-4.90	< .001	< .001
	MVPA	0.10	0.05	736	1.97	< .05	.092
	SE:MVPA	0.08	0.07	736	1.07	.287	.385
VPA	SE	-0.35	0.08	590	-4.62	< .001	< .001
	VPA	0.12	0.05	590	2.23	< .05	.056
	SE:VPA	0.10	0.08	590	1.27	.203	.304
MPA	SE	-0.32	0.07	693	-4.65	< .001	< .001
	MPA	0.05	0.05	693	0.98	.325	.428
	SE:MPA	0.08	0.07	693	1.04	.297	.396
ACC-MVPA	SE	-0.47	0.09	394	-5.13	< .001	< .001
	ACC-MVPA	0.22	0.07	394	3.29	< .01	< .01
	SE:ACC-MVPA	-0.03	0.10	394	-0.32	.748	.838
ACC-VPA	SE	-0.48	0.11	295	-4.58	< .001	< .001
	ACC-VPA	0.12	0.08	295	1.59	.113	.187
	SE:ACC-VPA	0.15	0.11	295	1.33	.184	.287
ACC-MPA	SE	-0.47	0.09	394	-5.14	< .001	< .001
	ACC-MPA	0.24	0.07	394	3.49	< .001	< .01
	SE:ACC-MPA	-0.08	0.10	394	-0.82	.414	.525
ACC-LPA	SE	-0.47	0.09	394	-5.16	< .001	< .001
	ACC-LPA	0.18	0.06	394	2.77	< .01	< .05
	SE:ACC-LPA	0.10	0.10	394	1.09	.275	.384
ACC-ENMO	SE	-0.46	0.09	394	-5.07	< .001	< .001
	ACC-ENMO	0.21	0.07	394	3.15	< .01	< .01
	SE:ACC-ENMO	0.07	0.10	394	0.75	.456	.565

Note. MVPA moderate-vigorous physical activity; VPA vigorous physical activity; MPA moderate physical activity; LPA light physical activity; ENMO Euclidean norm minus one; ACC accelerometry; SE stressful experience occurred (= yes); *p.unadj* unadjusted *p*-value.

Supplementary Table 4

Results of multilevel models testing the associations of the occurrence of a stressful experience during the day various physical activity variables, and an interaction of both with negative affect in the evening

	Predictor	Beta	*SE*	*df*	*t*	*p.unadj*	*p*
MVPA	SE	0.34	0.07	736	5.08	< .001	< .001
	MVPA	-0.15	0.05	736	-3.04	< .01	< .01
	SE:MVPA	-0.04	0.07	736	-0.54	.588	.691
VPA	SE	0.38	0.08	590	5.12	< .001	< .001
	VPA	-0.13	0.05	590	-2.34	< .05	< .05
	SE:VPA	-0.09	0.08	590	-1.18	.237	.339
MPA	SE	0.32	0.07	693	4.64	< .001	< .001
	MPA	-0.12	0.05	693	-2.23	< .05	.056
	SE:MPA	-0.01	0.07	693	-0.14	.892	.930
ACC-MVPA	SE	0.49	0.09	394	5.27	< .001	< .001
	ACC-MVPA	-0.03	0.07	394	-0.40	.688	.786
	SE:ACC-MVPA	-0.20	0.10	394	-2.09	< .05	.070
ACC-VPA	SE	0.50	0.11	295	4.62	< .001	< .001
	ACC-VPA	-0.14	0.08	295	-1.78	.076	.135
	SE:ACC-VPA	-0.14	0.11	295	-1.26	.210	.311
ACC-MPA	SE	0.49	0.09	394	5.25	< .001	< .001
	ACC-MPA	-0.01	0.07	394	-0.17	.868	.921
	SE:ACC-MPA	-0.20	0.10	394	-2.02	< .05	.083
ACC-LPA	SE	0.49	0.09	394	5.43	< .001	< .001
	ACC-LPA	-0.07	0.06	394	-1.08	.282	.385
	SE:ACC-LPA	-0.27	0.10	394	-2.79	< .01	< .05
ACC-ENMO	SE	0.48	0.09	394	5.28	< .001	< .001
	ACC-ENMO	-0.09	0.07	394	-1.31	.191	.291
	SE:ACC-ENMO	-0.21	0.10	394	-2.14	< .05	.066

Note. MVPA moderate-vigorous physical activity; VPA vigorous physical activity; MPA moderate physical activity; LPA light physical activity; ENMO Euclidean norm minus one; ACC accelerometry; SE stressful experience occurred (= yes); *p.unadj* unadjusted *p*-value.

Supplementary Table 5

Simple slopes for the association of various physical activity variables with affective states for days on which no stressful experience occurred vs. on which they occurred

Outcome	PA intensity	SE	Beta	*SE*	*t*	*p.unadj*	*p*
Positive affect	MVPA	No	0.10	0.05	1.97	< .05	.092
		Yes	0.17	0.05	3.30	< .01	< .01
	VPA	No	0.12	0.05	2.23	< .05	.056
		Yes	0.22	0.06	3.72	< .001	< .01
	MPA	No	0.05	0.05	0.98	.325	.428
		Yes	0.13	0.05	2.39	< .05	< .05
	ACC-MVPA	No	0.22	0.07	3.29	< .01	< .01
		Yes	0.19	0.07	2.85	< .01	< .05
	ACC-VPA	No	0.12	0.08	1.59	.113	.187
		Yes	0.27	0.08	3.42	< .001	< .01
	ACC-MPA	No	0.24	0.07	3.49	< .001	< .01
		Yes	0.16	0.07	2.35	< .05	< .05
	ACC-LPA	No	0.18	0.06	2.77	< .01	< .05
		Yes	0.28	0.07	4.03	< .001	< .001
	ACC-ENMO	No	0.21	0.07	3.15	< .01	< .01
		Yes	0.28	0.07	4.06	< .001	< .001
Negative affect	MVPA	No	-0.15	0.05	-3.04	< .01	< .01
		Yes	-0.19	0.05	-3.58	< .001	< .01
	VPA	No	-0.13	0.05	-2.34	< .05	< .05
		Yes	-0.22	0.06	-3.69	< .001	< .01
	MPA	No	-0.12	0.05	-2.23	< .05	.056
		Yes	-0.13	0.05	-2.33	< .05	< .05
	ACC-MVPA	No	-0.03	0.07	-0.40	.688	.786
		Yes	-0.23	0.07	-3.37	< .001	< .01
	ACC-VPA	No	-0.14	0.08	-1.78	.076	.135
		Yes	-0.28	0.08	-3.50	< .001	< .01
	ACC-MPA	No	-0.01	0.07	-0.17	.868	.921
		Yes	-0.21	0.07	-3.04	< .01	< .01
	ACC-LPA	No	-0.07	0.06	-1.08	.282	.385
		Yes	-0.34	0.07	-4.79	< .001	< .001
	ACC-ENMO	No	-0.09	0.07	-1.31	.191	.291
		Yes	-0.29	0.07	-4.23	< .001	< .001

Note. PA physical activity; MVPA moderate-vigorous physical activity; VPA vigorous physical activity; MPA moderate physical activity; LPA light physical activity; ENMO Euclidean norm minus one; ACC accelerometry; SE stressful experience; *p.unadj* unadjusted *p*-value.

Supplementary Table 6

Results of multilevel models testing the associations of stress intensity of stressful experiences during the day, various physical activity variables, and an interaction of both with positive affect in the evening (H2b)

	Predictor	Beta	*SE*	*df*	*t*	*p.unadj*	*p*
MVPA	SI	-0.29	0.06	338	-5.17	< .001	< .001
	MVPA	0.15	0.05	338	2.79	< .01	< .05
	SI:MVPA	0.03	0.06	338	0.54	.592	.691
VPA	SI	-0.32	0.06	258	-4.89	< .001	< .001
	VPA	0.19	0.06	258	3.05	< .01	< .01
	SI:VPA	0.06	0.08	258	0.79	.432	.542
MPA	SI	-0.29	0.06	327	-4.97	< .001	< .001
	MPA	0.12	0.06	327	2.25	< .05	.056
	SI:MPA	0.04	0.06	327	0.67	.505	.608
ACC-MVPA	SI	-0.29	0.08	177	-3.80	< .001	< .01
	ACC-MVPA	0.18	0.07	177	2.49	< .05	< .05
	SI:ACC-MVPA	0.07	0.08	177	0.93	.355	.464
ACC-VPA	SI	-0.26	0.09	127	-2.86	< .01	< .05
	ACC-VPA	0.23	0.08	127	2.94	< .01	< .05
	SI:ACC-VPA	0.10	0.08	127	1.24	.219	.320
ACC-MPA	SI	-0.30	0.08	177	-3.80	< .001	< .01
	ACC-MPA	0.16	0.07	177	2.23	< .05	.056
	SI:ACC-MPA	0.06	0.08	177	0.82	.415	.525
ACC-LPA	SI	-0.30	0.08	177	-3.99	< .001	< .001
	ACC-LPA	0.28	0.07	177	3.86	< .001	< .001
	SI:ACC-LPA	0.15	0.09	177	1.70	.090	.155
ACC-ENMO	SI	-0.28	0.08	177	-3.73	< .001	< .01
	ACC-ENMO	0.25	0.07	177	3.51	< .001	< .01
	SI:ACC-ENMO	0.10	0.08	177	1.33	.185	.287

Note. MVPA moderate-vigorous physical activity; VPA vigorous physical activity; MPA moderate physical activity; LPA light physical activity; ENMO Euclidean norm minus one; ACC accelerometery; SI stress intensity; *p.unadj* unadjusted *p*-value.

Supplementary Table 7

Results of multilevel models testing the associations of stress intensity of stressful experiences during the day, various physical activity variables, and an interaction of both with negative affect in the evening (H2b)

	Predictor	Beta	*SE*	*df*	*t*	*p.unadj*	*p*
MVPA	SI	0.34	0.05	338	6.17	< .001	< .001
	MVPA	-0.16	0.05	338	-3.13	< .01	< .01
	SI:MVPA	-0.02	0.06	338	-0.40	.689	.786
VPA	SI	0.37	0.06	258	5.86	< .001	< .001
	VPA	-0.18	0.06	258	-3.09	< .01	< .01
	SI:VPA	-0.10	0.07	258	-1.43	.154	.245
MPA	SI	0.34	0.06	327	6.08	< .001	< .001
	MPA	-0.11	0.05	327	-2.14	< .05	.066
	SI:MPA	-0.02	0.06	327	-0.30	.762	.845
ACC-MVPA	SI	0.38	0.07	177	5.18	< .001	< .001
	ACC-MVPA	-0.23	0.07	177	-3.38	< .001	< .01
	SI:ACC-MVPA	-0.02	0.07	177	-0.23	.817	.890
ACC-VPA	SI	0.39	0.09	127	4.38	< .001	< .001
	ACC-VPA	-0.23	0.08	127	-2.84	< .01	< .05
	SI:ACC-VPA	-0.04	0.08	127	-0.46	.646	.749
ACC-MPA	SI	0.39	0.07	177	5.24	< .001	< .001
	ACC-MPA	-0.23	0.07	177	-3.35	< .001	< .01
	SI:ACC-MPA	0.00	0.07	177	-0.02	.981	.986
ACC-LPA	SI	0.39	0.07	177	5.44	< .001	< .001
	ACC-LPA	-0.34	0.07	177	-4.91	< .001	< .001
	SI:ACC-LPA	-0.09	0.08	177	-1.08	0.283	.385
ACC-ENMO	SI	0.36	0.07	177	4.96	< .001	< .001
	ACC-ENMO	-0.27	0.07	177	-3.91	< .001	< .001
	SI:ACC-ENMO	-0.03	0.08	177	-0.34	.736	.829

Note. MVPA moderate-vigorous physical activity; VPA vigorous physical activity; MPA moderate physical activity; LPA light physical activity; ENMO Euclidean norm minus one; ACC accelerometery; SI stress intensity; *p.unadj* unadjusted *p*-value.
